# Improvement of Oxidative Status, Milk and Cheese Production, and Food Sustainability Indexes by Addition of Durum Wheat Bran to Dairy Cows’ Diet

**DOI:** 10.3390/ani9090698

**Published:** 2019-09-18

**Authors:** Adriana Bonanno, Antonino Di Grigoli, Massimo Todaro, Marco Alabiso, Francesca Vitale, Adriana Di Trana, Daniela Giorgio, Luca Settanni, Raimondo Gaglio, Barbara Laddomada, Giuseppe Di Miceli

**Affiliations:** 1 Department of Agricultural, Food and Forest Sciences (SAAF), University of Palermo, Viale delle Scienze n. 13, 90128 Palermo, Italy; adriana.bonanno@unipa.it (A.B.); massimo.todaro@unipa.it (M.T.); marco.alabiso@unipa.it (M.A.); francy.vitale88@hotmail.it (F.V.); luca.settanni@unipa.it (L.S.); raimondo.gaglio@unipa.it (R.G.); giuseppe.dimiceli@unipa.it (G.D.M.); 2 School of Agricultural, Forestry, Food and Environmental Sciences (SAFE), Viale dell’Ateneo Lucano n. 10, 85100 Potenza, Italy; adriana.ditrana@unibas.it (A.D.T.); giorgio_daniela@alice.it (D.G.); 3 Institute of Sciences of Food Production (ISPA), National Research Council (CNR), Via Monteroni, 73100 Lecce, Italy; barbara.laddomada@ispa.cnr.it

**Keywords:** durum wheat bran, by-products, phenolic acids, antioxidant power, human-edible feed conversion efficiency, dairy products

## Abstract

**Simple Summary:**

In the near future, the expected increase in world population will enhance feed versus food competition between animals and humans to face the increasing demand by humans. For this reason, it is of paramount importance to feed ruminants with sources alternative to those representing themselves foods for humans. Durum wheat bran (DWB), similar to other by-products of the agri-food industry, can meet this need, its fiber content is high and represents also a remarkable source of phenolic acids, especially ferulic acid. This compound exerts antioxidant properties improving the health status of animals, and allows the production of functional foods more and more requested by consumers. Accordingly, this experiment demonstrated the suitability of using DWB for dairy cows feeding, because it led to clear advantages in terms of oxidative status of animals, quality of dairy products, and feeding costs. Furthermore, DWB improved the human-edible feed conversion efficiency reducing the use of human-edible feed.

**Abstract:**

Durum wheat bran (DWB) is a by-product mostly used in feeding ruminants, contributing to decrease in the utilization of feeds suitable as foods for human consumption, thus improving the sustainability of livestock production. However, the potential benefits of DWB, due to its content in phenolic acids, mainly consisting of ferulic acid with antioxidant properties, have not been well clarified yet. Accordingly, in this experiment, 36 lactating cows divided into three groups received, over a period of 100 days, one of three concentrates including DWB at 0% (DWB0), 10% (DWB10), or 20% (DWB20). The concentrates were formulated to be isoproteic and isoenergetic and, to balance the higher fiber content of the concentrates with DWB, the hay in the diets was slightly reduced. During the trial, the group feed intake and the individual milk production were monitored, and cheese was made with bulk milk from each group. Milk yield and microbiological characteristics of milk and cheese were similar among groups, indicating no DWB effect on cows performance and fermentation process. Milk from DWB20 group resulted slightly higher in casein and curd firmness (a_2r_). In cows fed DWB, the higher polyphenol intake was responsible for higher blood contents of these bioactive compounds, that seemed to have contributed in reducing the level of reactive oxygen metabolites (ROMs), which were higher in DWB0 cows. DWB20 cheeses showed a higher polyphenol content, lower number of peroxides, and higher antioxidant capacity than DWB0 cheeses. DWB20 and DWB10 diets resulted less expensive. In addition, the DWB20 group showed the best indexes heFCE (human edible feed conversion efficiency = milk/human edible feed) and NFP (net food production = milk − human edible food), expressed as crude protein or gross energy. In conclusion, the DWB fed to dairy cows at 12% of diet dry matter (DM) can lead to benefits, such as the improvement of oxidative status of cows, milk quality, shelf-life, and functional properties of cheese, and might contribute to reduce the feeding cost and limit the human-animal competition for feeding sources.

## 1. Introduction

In the next years, the expected consistent increase in world’s population will lead to high rates of increasing demands of animal products and, consequently, a greater proportion of crop grains should be destined to feeding animals.

Today, intensive livestock systems are much censured because, as is known, ruminants use, with scarce efficiency, many feeds such as corn and soybean grains that can be used directly by humans. Some researchers have calculated that almost 90% of the calories produced by vegetable crops used in intensive farming are lost and are not transferred to animal products [1]. This suggests that using human-edible crops to feed animals is an inefficient way to provide calories to humans, and also it conflicts with the challenges around global sustainability. Consequently, facing with the increasing demand of food for the world’s growing population, the use of non-edible products for humans, mostly crop by-products, as feeds for livestock animals would provide a net gain of foods of high biological value for humans. Durum wheat bran (DWB) can be counted among this type of feed because, due to the high fiber content, this energy source has low efficiency for humans [2].

DWB, a by-product of the wheat milling industry, is a valid ingredient for ruminants feeding, both for its chemical composition and because it allows a reduction in feeding costs favoring the exploitation of local resources [3]. In southern Italy, where the cultivation of durum wheat is widespread [4], DWB represents an important feed resource because it is largely available and can be found locally [5]. Furthermore, the low price encourages its use, especially when the prices of other alternative ingredients increase. The high fiber content, the high-quality proteins, and the antioxidant activity of DWB can be of particular interest for its use in ruminant rations. As matter of fact, ruminants can take better advantages of DWB than monogastric species, because they are able to use fiber-rich feeds more efficiently, making available protected proteins. Thus, ruminants can benefit from improved conditions for the ruminal ecosystem [6], which instead could be compromised by concentrates with high and rapid rumen degradability.

DWB is also an important source of ferulic acid, a phenolic acid with positive health benefits [7,8,9]. The use of bioactive compounds such as polyphenols in animal diet represents a natural way to improve the oxidative status, health, and performance of animals, as well as to allow the production of functional foods for human consumption. Several studies focused on the use of plants and their extracts to improve the antioxidant status of animals [10,11,12,13,14] and provide healthy products for human consumers. The latter aspect is of relevance because modern consumers are more conscious of the nutritional and functional value of foods and their role in human health.

For these reasons, the aims of this study were to test the inclusion of DWB in the rations of lactating dairy cows in order to evaluate: (i) the improvement of nutrient utilization of animals as well as their oxidative protection, (ii) the production of milk and cheese of high healthy value for consumers thanks to their chemical characteristics and microbiological profile, (iii) the advantages deriving from the increased livestock sustainability from the economic and the social perspectives.

## 2. Materials and Methods

### 2.1. Animals, Feeding Systems, and Management

This experiment was conducted in a dairy farm located 726 m above sea level in the province of Palermo (Sicily, Italy, 37°42′ N, 14°08′ E), for 100 days from mid-January to end-April.

A total of 36 Italian Red Pied lactating cows, initially 92 ± 56 days in milk (DIM), 2.9 ± 1.6 parity and producing 28.2 ± 6.2 kg/day of milk, were divided into three homogeneous groups of 12 cows each and assigned to feeding regime differing exclusively for the content of DWB in the concentrate, according to the following scheme: (i) control, 0% of DWB (DWB0), (ii) 10% of DWB, corresponding to 1.5 kg/day per cow (DWB10), (iii) 20% of DWB, corresponding to 3.0 kg/day per cow (DWB20). The three groups were kept in separated areas within a free-stall building with straw-bedded cubicles as a resting area. Each group had access to its feeding area to receive, twice per day, a total mixed ration (TMR) composed of hay and one of the three different concentrates. 

The concentrates used in the trial were formulated to be isoproteic and isoenergetic. The composition of the TMRs are reported in Table 1. Due to the high fiber content of DWB, the chemical composition of TMRs were balanced with lower amount of hay for DWB10 and DWB20 groups. 

All cows involved in the trial had previous experience with free-stall housing and during the research, they showed no sign of illness. Cows were managed according to the Directive 2010/63/EU on the protection of animals used for scientific purposes and the trial complied with the Italian legislation on animal care (DL n. 26, 4 March 2014).

### 2.2. Measurement, Sampling, and Analytical Methods

#### 2.2.1. Feeds

During the trial, the residual TMR amounts of the three groups were weighed twice a week to calculate the feed intake. The feed samples (hay and concentrates) were taken weekly and analysed according to the AOAC methods [15] to determine dry matter (DM), crude protein (CP), ether extract and ash. The fibrous fractions, as aNDFom (neutral detergent fiber using heat-stable amylase and exclusive of residual ash), ADFom (ash-free acid detergent fiber), and ADL (acid detergent lignin), were determined in accordance to the AOAC methods [15] and Van Soest et al. [16]. Non structural carbohydrates (NSC) content was calculated as follows: 100 − (CP + Ether extract + Ash + aNDFom). The net energy for lactation (Mcal/kg DM) of the feeds was estimated using the National Research Council equations [17]. 

Concentrates and DWB were also analysed for the content of individual phenolic acids, which were extracted from 250 mg of each sample according to Laddomada et al. [18]. Briefly, the extraction started from sample delipidation, followed by alkaline hydrolysis, acidification and recovery in ethyl acetate. The extracts were dried under nitrogen flux, re-dissolved in 200 µL of 80:20 methanol/water and quali-quantitatively analysed using an Agilent 1100 Series HPLC-DAD system (Agilent Technologies, Santa Clara, CA, USA). The separation occurred under the following conditions: a reversed-phase C18 (2) Luna column (Phenomenex, Torrance, CA, USA) (5 mm, 250 × 4.6 mm), column temperature of 30 °C, water-acetonitrile-acetic acid (88:10:2, v/v/v) as mobile phase, flow rate of the mobile phase of 1 mL/min, 20 μL injection volume. The peaks were identified by comparing the corresponding retention times and UV-Vis spectra to those of authentic phenolic acid standards, and quantified based on their maximum UV-Vis absorption, and using calibration curves of phenolic acid standards. Authentic standards of phenolic acids were obtained from Sigma-Aldrich (Gillingham, UK) and included p-hydroxybenzoic acid, vanillic acid, syringic acid, p-coumaric acid, sinapic acid, and ferulic acid. All standards were prepared as stock solutions at 2 mg/mL in 80:20 v/v ethanol/water which were stored in the dark at −20 °C. Each sample was analysed in triplicate and the concentrations of individual phenolic acids were expressed in μg/g DM.

The chemical composition of the feeds used in the diets is reported in Table 2. The feed intake data of each group were used to calculate the average daily intake of CP, aNDFom, ADFom, ADL, NFC, NE_L_, and phenolic acids.

#### 2.2.2. Milk and Cheese

The cows were milked mechanically twice a day, at 5:00 h and at 17:00 h. Individual milk yield was weighed and sampled six times during the trial at 0, 14, 35, 56, 77, and 100 days. Individual milk samples were analysed for lactose, fat, protein, casein, and somatic cell count (SCC) using infrared methods (Combifoss 6000, Foss Electric, Hillerød, Denmark), and urea by enzymatic methods using the difference in pH (CL-10 Plus, Eurochem, Rome, Italy). The values of pH and titratable acidity were measured by a pH meter (HI 9025, Hanna Instruments Inc., Ann Arbor, MI, USA) and as Soxhlet and Henkel degrees (°SH/50 mL), respectively. Total nitrogen (TN), non-casein nitrogen (NCN), and non-protein nitrogen (NPN) were determined according to standard FIL-IDF (Fédération Internationale du Lait - International Dairy Federation) procedures [19,20]. Individual milk samples were evaluated for clotting ability by measuring coagulation time (r, min), curd-firming time (k_20_, min), curd firmness (a_30_, mm), and curd firmness after twice the coagulation time (a_2r_, mm) with a Formagraph instrument (Formagraph, Foss Electric, Padova, Italy).

On the same days of milk yield recording, 3.5 kg of bulk milk was collected from each group and subjected to a cheese-making process. Each refrigerated (4 °C) sample of raw and whole milk, contained in a pyrex glass beaker, was heated by immersion in a water bath for about 30 min to reach 37 °C. After addition of 8.75 mL calf liquid rennet (1:15,000, 80 ± 5% chymosin, and 20 ± 5% pepsin, Chr. Hansen, Parma, Italy) diluted in distilled water (1.6:100), milk was kept at 37 °C until coagulation, which occurred approximately after 1 h. The curd was broken to the dimension of rice grains. Each curd was cooked at 80 °C for 4 min in a water bath, removed from the beaker and pressed with hands into a cylindrical, perforated plastic mould to drain the whey, and turned every 5 min to facilitate draining. After 15 min, each mould was held in the water bath at 60 °C for 1 h. Then the cheeses were placed on a flat surface for draining and, after 1 h, transferred to a cold room for 30 days at 16 °C and 80% of relative humidity.

Cheeses were sampled after 30 days and analysed according to the standard FIL-IDF methods for the determination of DM [21], protein [22], fat [23], and ash [24]. Cheese extracts were prepared according to the method described by Rashidinejad et al. [25] in order to measure total phenolic compound content using the Folin-Ciocalteau colorimetric method [26]. The results are expressed as mg of gallic acid equivalent (GAE)/g DM. The oxidation status of fat was assessed in the cheese samples determining the peroxide value (mEq O_2_/kg fat) as index of primary lipid oxidation [27]. Moreover, the thiobarbituric acid–reactive substances (TBARS) were determined as a measure of the secondary lipid oxidation products and expressed as μg malonylaldehyde (MDA)/kg DM, according to the method proposed by Tarladgis et al. [28] and modified by Mele et al. [29]. Finally, the total antioxidant capacity (TAC) of the cheeses was measured using the ferric ion reducing antioxidant power (FRAP) assay [30].

#### 2.2.3. Microbiological Analyses of Milk and Cheese, LAB Isolation and Phenotypic Grouping 

Microbiological analyses were conducted by plate counts for the main microbial groups in milk and cheeses. Milk samples were subjected to decimal serial dilutions in Ringer’s solution (Oxoid, Milan, Italy), while twenty-five g of cheese samples were first transferred into sterile plastic bags (BagLight^®^ 400 Multilayer^®^ bags, Interscience, Saint Nom, France), added with 225 mL of sodium citrate (2% w/v) solution (Sigma Aldrich, Milan, Italy), homogenised in a stomacher (Bag-Mixer 400, Interscience, Saint Nom, France) for 2 min at the maximum speed, and then serially diluted with Ringer’s solution.

The inoculation, cultivation, and incubation of the different microbial groups were as follows: total mesophilic microorganisms (TMM) were spread plated on plate count agar (PCA) supplemented with 1 g/L skimmed milk and incubated aerobically for 72 h at 30 °C, total psychrotrophic microorganisms (TPM) were plated as described for TMM, but incubation was performed for 7 days at 7 °C, mesophilic and thermophilic rod-shaped lactic acid bacteria (LAB) were pour plated on de Man-Rogosa-Sharpe (MRS) agar, acidified to pH 5.4 with lactic acid (5 mol/L) and incubated anaerobically for 48 h at 30 °C and 44 °C, respectively, mesophilic and thermophilic LAB cocci were pour plated on Medium 17 (M17) agar and incubated anaerobically for 48 h at 30 °C and 44 °C, respectively, enterococci were spread plated on kanamycin esculin azide (KAA) agar and incubated aerobically for 24 h at 37 °C, members of the *Enterobacteriaceae* family were pour plated on double-layered violet red bile glucose agar (VRBGA) and incubated aerobically for 24 h at 37 °C. Anaerobiosis occurred in hermetically sealed jars added with the AnaeroGen AN25 system (Oxoid, Milan, Italy). Microbiological analyses were carried out in duplicate. All media were purchased from Oxoid.

After incubation, for each morphology (shape, surface, color, margin, and elevation) five identical colonies were randomly collected from MRS and M17 and transferred to the corresponding broth media. Bacterial isolates were subjected to the purification procedure after several consecutive subcultures and the cell homogeneity was verified by an optical microscope (Zeiss, Oberkochen, Stuttgart, Germany). Gram-positive (determined by KOH method) and catalase-negative (determined with addition of 5%, v/v, H_2_O_2_ to each colony) bacterial cultures, presumed to be LAB, were further characterized phenotypically and grouped for their morphological, physiological, and biochemical characteristics. 

Cell morphology and disposition were evaluated by microscopic inspection. Growth at 15 °C and 45 °C, heat resistance (60 °C for 30 min), NH_3_ production from arginine, esculin hydrolysis, acid production from carbohydrates, and CO_2_ production from glucose were determined as previously reported by Gaglio et al. [31]. The coccus-shaped isolates were further grouped according to their growth at pH 9.2 and in the presence of 6.5 g/liter NaCl to separate enterococci able to grow under these conditions from other LAB.

#### 2.2.4. Blood Analysis

To assess the oxidative status of cows, blood samples were taken at 0, 14, and 100 days. Individual blood samples from fasted cows were collected in the morning by coccygeal venipuncture. Each blood sample was divided into two aliquots and transferred into two vacutainer tubes (Vacuette, Greiner Bio-One, Kremsmunster, Austria), one containing lithium heparin instantly placed on ice and the other one with no anticoagulant. Plasma was obtained by centrifugation at 3000 rpm for 10 min at 4 °C. Serum samples were obtained by maintaining the blood samples at room temperature until clotting, which was followed by centrifugation at 3000 rpm for 7 min. Plasma and serum samples were stored at −80 °C until analyses were performed. Gamma globulin (IgG, Bethyl Diagnostics) were determined using the ELISA(Enzyme Linked Immuno-Sorbent Assay) technique with commercial kits. Plasma oxidants and antioxidant capacity were measured with two different commercial kits (Diacron, Grosseto, Italy) by determining reactive oxygen metabolites (ROMs), mainly hydroperoxides generated by the oxidation of biomolecules, and biological antioxidant potential (BAP), measuring the capacity of plasma samples to reduce iron from ferric (Fe^3+^) to ferrous (Fe^2+^) form. The results of the d-ROMs test were expressed as arbitrary units called Carratelli units’ (U. Carr.), where 1 U. Carr. corresponds to 0.08 mg/100 mL H_2_O_2_, whereas the results of the BAP test were expressed in μEq/L reduced iron. The oxidative stress index (OSI) was calculated using the formula log BAP/ROMs × 100, proposed by Ranade et al. [32]. 

Plasma samples were also subjected to the determination of total polyphenols, using the Folin-Ciocalteu method [26], after extraction according to Serafini et al. [33], of free polyphenols with the Folin-Ciocalteu method and after protein precipitation according to the method described by Santiago-Arteche et al. [34], of conjugated polyphenols by difference between the previous two classes.

#### 2.2.5. Feed Efficiency Index 

Feed intake data were used for the calculation of human-edible feed conversion efficiency (heFCE), defined as human-edible output in the form of animal products (in this research constituted by milk) divided by potential human-edible input via feedstuffs as kg crude protein (CP) and MJ gross energy (GE), as proposed by Ertl et al. [35]:

heFCEcp = kg milk CP/kg CP of human − edible feed

heFCEge = MJ milk GE/MJ GE of human − edible feed

The human-edible proportion estimated by Wilkinson et al. [2] was used to calculate the human-edible input of the different ingredients constituting the three TMRs used in the trial. The energy content of milk was calculated applying the formula defined by NRC [17], including milk protein, fat, and lactose with the factor 4.184 to convert calories to joules. 

Finally, the net food production (NFP) was calculated in terms of g/day CP and MJ/day GE for the three experimental diets, subtracting the human edible input to human edible output (milk) [35]:

NFPcp (g/day) = g/day milk CP − g/day CP of human edible food

NFPge (MJ/day) = MJ/day milk GE − MJ/day GE of human edible food

### 2.3. Statistical Analysis

Data were analysed statistically using the software SAS (Version 9.2, SAS Institute Inc., Campus Drive Cary, NC, USA).

For individual data of milk production and blood analysis, with the cow as the experimental unit, the fixed effects of diet (D, three levels: DWB0, DWB10, DWB20), sampling day (SD, five levels for milk production: 14, 35, 56, 77, and 100 days, two levels for blood samples: 14 and 100 days) and their interaction D×SD were assessed using a MIXED model for repeated measures, with SD as the repeated measure, and cow as the repeated subject, regarded as a random error term. The pre-experimental milk production and blood analysis data (day 0) were used as covariates only when significant. SCC values were transformed logarithmically (log_10_) before analysis. When the effects of SD and D×SD were not significant, the corresponding means were not reported in the tables.

Data of feed intake, milk and cheese microbiological profile, and cheese composition were subjected to GLM procedure, with the group as experimental unit, evaluating only the effect of diet. When a statistically significant (*p* value ≤ 0.05) effect was detected, the means were compared by Tukey’s multiple range test at 0.01 and 0.05 *p* levels.

## 3. Results and Discussion

### 3.1. Feed Intake

The chemical composition of feeds used in the trial is shown in Table 2. The aNDFom and ether extract content in the concentrates increased with DWB level, due to the higher amount of structural carbohydrates and fat of DWB than other ingredients. Despite these differences, all concentrates were comparable in terms of energy and protein value.

Although phenolic acids are present in cereals, including corn and barley used to formulate the concentrates, their content strongly increased with the inclusion of DWB, due to its high concentration in these bioactive compounds. Among phenolic acids, ferulic acid was found at the highest concentration.

Table 3 shows the feed intake of cows during the study. 

The groups fed with DWB, due to the composition of TMRs which was balanced for the fiber content, consumed less hay and a slightly higher amount of concentrate. The total DM, CP, and NFC intakes for the DWB0 and DWB10 groups were registered at higher levels than that observed for DWB20 group. The intake of the fibrous components, especially ADFom and ADL, was different among groups. Nevertheless, the variations were negligible, due to the low variability during the trial, and also because the TMRs were formulated to compensate for the higher amount of fiber in the DWB reducing the hay content. The intake in terms of net energy was slightly different between groups, with an average value of 140.5 MJ/day per cow. As expected, the ingestion of phenolic acids with the concentrate was higher for DWB20 group, followed by DWB10 group. However, this ingestion does not include the number of phenolic acids from hay. In this regard, data reported in literature are variable, for example, in alfalfa hay the number of phenolic acids can range from 1500 to 2700 µg/g DM [36,37,38]. In the present study, assuming a phenolic acid level of hay equal to that of DWB (2484 µg/g DM), and considering the recorded hay consumptions, the total intake of phenolic acids with the DWB-based diets would be higher than that without DWB addition (41.0, 47.3 and 47.1 g/day per head for DWB0, DWB10 and DWB20 diets, respectively).

### 3.2. Oxidative and Immune Status of Cows

Table 4 shows data on blood analysis. Overall, the presence of 10 or 20% of DWB in the diet increased significantly total, free, and conjugated polyphenols in the blood of cows. Nevertheless, DWB cows showed values of polyphenols almost comparable from 14 to 100 days of the experiment, whereas the control group showed an increase of conjugated polyphenols in the same temporal interval until reaching the same level of DWB cows at 100 days. This trend, which implied a significant interaction, could be explained assuming an increased availability of total polyphenols in the control diet, deriving from other feeds components than DWB, such as corn and barley. However, the transfer of polyphenols from feed to animals seemed to have occurred, even if only partially and independently of the amount ingested, as suggested by the same levels recorded with DWB10 and DWB20 concentrates. In their review, O’Connell and Fox [39] mention several experiments where the transfer of polyphenols from ingested feeds to animals occurs with frequency. 

The ROMs were significantly affected by diet, sampling day, and their interaction. The values of ROMs were significantly lower in the groups fed with DWB, and increased from 14 to 100 days in DWB0 and DWB10 groups, whereas their level did not change in the DWB20 group.

The ROMs, considered indicators of free radicals production, originate from metabolic reactions at cellular level and, if produced in excess or more quickly than their neutralization by the antioxidant mechanisms, they provoke a status of oxidative stress that contributes to arise metabolic disorders, interfering with physiological functions and productivity of livestock animals [40,41]. In this experiment, the increase of ROMs at the end of the trial was in contrast with previous findings reporting higher free radical generation from cows in the first phase of lactation [42,43]. An explanation of this discrepancy could be that, at the beginning of this trial, the cows were at about 100 DIM, a phase in which probably they did not suffer the stressful effect that occurs mainly in the post-partum period. However, it should be emphasized that the increase of ROMs did not occur until the end of the trial for the DWB20 group, that seems to take advantage of the higher dose of DWB. The introduction of DWB at 20% in the concentrate might have contributed to preventing the ROMs increase in the cows, confirming that nutrition plays a defining role in the oxidative status of farmed animals [44].

The BAP was not influenced by dietary treatment, as also previously observed, [45], but showed a positive increase after 100 days of trial in all groups. On the contrary, OSI, given by the ratio between BAP and ROMs, was positively increased in DWB-fed cows compared to the control group, although a more evident improvement was recorded by DWB20 cows at 100 days, explaining the significant interaction D*SD. The OSI showed the same trend observed for ROMs in the different periods. Therefore, the values of ROMs and OSI were positively influenced by diets with higher level of wheat bran, in particular at the end of the trial, after a long period of feed administration. These effects can be linked to the antioxidant action exerted by polyphenols, and especially ferulic acid, due to their particular structure which is capable of binding oxygen free radicals and their transformation into compounds without reactivity [46]. This phenomenon is due to the presence of electron donor groups on the benzene ring of phenolic acids, which allow the termination of the chain reactions triggered by free radicals, and to the presence of the carboxyl group that together with the double unsaturated bond provide additional attack sites for free radical inactivation [47]. Moreover, it is also possible that the improvement of rumen conditions due to feeding wheat bran rich in fiber contributed to the reduced production of free radicals by cows [44].

The improvement of oxidative stress status probably affected the immune system of DBW20 cows. Indeed, at the end of the trial, DWB20 cows showed higher levels of immunoglobulins (IgG) than DWB0 cows, with intermediate values registered for DWB10 cows (Table 4), indicating a high efficiency of the immune system in the intermediate-final phase of lactation. There is ample evidence in the literature regarding the improvement in the immune and health status of animals when they receive feeds rich in polyphenols [48,49].

### 3.3. Dairy Products

Milk yield and composition are shown in Table 5. 

The dietary treatment did not affect the milk yield, in agreement with recent researches on the use of wheat bran and other by-products in cows’ diet [35,50,51], confirming that these balanced concentrate formulations do not modify milk production. Small differences (*p* < 0.10) among groups emerged exclusively for milk protein and casein percentages, which were slightly higher in the DWB20 group (*p* < 0.10). These differences could be imputable to improved efficiency of dietary protein utilization by cows fed with the highest amount of wheat bran. Since the DWB20-fed cows showed a remarkable increase in the intake of phenolic compounds, these substances could have contributed to a reduction of the activity of protein-degrading microorganisms of the rumen, and a consequent increase of rumen un-degraded protein and amino acids absorbed in the intestine, thus enhancing their availability for the synthesis of milk protein. Similarly, an increase of milk casein content was observed by Bonanno et al. [52,53] as result of diets based on forage rich in polyphenolic molecules, such as condensed tannins. The curd firmness at twice of coagulation time (a_2r_) of DWB20 milk was higher than that of milk from the other groups, due to its higher casein content, in accordance with Cassandro et al. [54]. The chemical composition of the cheeses (Table 6) was not affected by dietary treatment. However, the DWB20 cheeses showed a higher total polyphenol content than the other cheeses, although the difference was not statistically significant (*p* > 0.10). The higher polyphenol content had positive effects on DWB20 cheese, characterized by a lower number of peroxides and a higher content of antioxidant substances measured by TAC than the other cheeses. Among cheese components, the fatty acids and, in particular, the polyunsaturated fatty acids are more prone to oxidation. Moreover, polyunsaturated fatty acids are considered of higher value than saturated fatty acids for human health, therefore, the preservation of their structure contributes not only to improve shelf-life, but also to increase the nutritional characteristics of cheeses [39]. In accordance with other studies [55,56,57], these results suggest that the polyphenols, and in particular ferulic acid, administered with the DWB diet have been transferred, although partially, to the cheeses, and they played a positive role for protecting the cheese fatty acids from the oxidation damage. 

### 3.4. Microbial Levels in Milk and Cheese

The cheeses produced in this study were made from raw cows’ milk without the addition of starter cultures, thus, curd acidification, as well as cheese ripening, relied exclusively on the indigenous milk microbial populations, since transformation occurred at laboratory levels in controlled conditions, excluding any microbiological contamination by the transformation. Cheese cannot be made without the activity of LAB [58]. For this reason, the microbiological analyses included the counts of viable populations and the isolation and characterization of LAB groups associated to milk and cheese. This approach was necessary to investigate the effect of cows’ diet on cheese microbiology. As matter of fact, polyphenols can exert an inhibitory effect on microorganisms depending on their chemical structure and concentration [59,60] and wheat bran is a source of polyphenolic compounds [61].

The levels of the different microbial groups investigated in this study are reported in Table 7. According to Tukey’s test, statistically significant differences were not found for any of the microbial groups’ objects of investigation among the three experimental groups (DWB0, DWB10 and DWB20) indicating that the different diets administered to cows did not influence the levels of microorganisms, in particular bacteria, in milk and cheese samples. In particular, the levels of TPM and TMM were around 6 Log CFU/mL in the three bulk milk samples and reached cell densities of 10^9^ CFU/g in all cheeses. The cell densities of TMM were almost equivalent to those of mesophilic LAB cocci in all samples analysed (both milk and cheese samples), showing that this bacterial group dominated the microbiotas of milk and were primarily responsible for the entire fermentation process of the cheese productions. Thermophilic LAB cocci increased their numbers consistently during fermentation and were found, on average, at levels slightly lower than those of mesophilic LAB cocci in cheese at 30 days of ripening. Mesophilic and thermophilic rod LAB showed an increase during fermentation, reaching levels of 10^7^ CFU/g in all cheese samples. The differences estimated between LAB cocci and rods during short-term ripening were previously reported for bovine cheeses [62,63] and are due to the different evolution of starter and non-starter LAB [58]. Generally, non-starter LABs are lactobacilli, which are characterized by a rod shape.

Enterococci were registered at approximately 5 log CFU/mL in raw milk and increased of about 3 log cycle in ripened cheeses demonstrating the ability of these bacteria to follow the different steps of cheese making and to persist during ripening [64]. Enterococci are strictly linked to the typicality of cheeses [65] and, for this reason, are being used as components of cheese adjunct cultures [66,67] or even as probiotics [68,69]. However, their presence is still controversial in raw milk cheeses due to the risks related to the transfer of antibiotic resistance genes to intestinal bacteria [70]. Our results closely agreed with those observed for other raw cows’ milk cheeses analysed at the same period of ripening [71]. 

The different milk and cheese samples were also evaluated for their hygienic characteristics. Members of the *Enterobacteriaceae* family were detected at around 10^5^ CFU/mL in all milk samples. Their levels showed a clear increase, estimated almost at 1 Log cycle higher in all ripened cheeses. Levels of these bacteria in the range 10^4^–10^5^ CFU/g are common in traditional raw cows’ milk cheeses ripened for barely 1–2 months [62,63,64,65,66,67,68,69,70,71,72,73]. However, the concentration of *Enterobacteriaceae* greatly decreases during ripening, since their growth is affected by the stressing cheese conditions [74].

### 3.5. Isolation and Grouping of LAB

A total of 464 colonies were collected from milk and cheese samples, which were microscopically separated into 325 cocci and 139 rods. After Gram and catalase tests, 248 cocci and 81 rods were still considered to be LAB cultures as being Gram-positive and catalase-negative. According to the combination of the phenotypic traits, the 329 presumptive LAB cultures were separated into 15 groups (Table 8). The highest number of groups (11) included cocci which presented two main cell dispositions: short chains (6 groups) and long chains (5 groups). Rod isolates formed four groups. The largest group (4) included almost 23% of the total isolates. Groups 1 and 2 were characterized by an obligate homofermentative metabolism for the lack of growth in the presence of pentose carbohydrates, while the other two rod groups showed an obligate heterofermentative metabolism. Furthermore, the isolates of the groups 1 and 11–15 showed the ability to grow at 45 °C but not at 15 °C and were classified as thermophilic LAB.

Although the levels of viable LAB determined by agar plate counts were not statistically different among cheeses, not all 15 phenotypic groups were found in the three experimental cheeses. In particular, two groups (10 and 12) that included less than ten isolates, representing a very low percentage of the pure cultures, were only found in control milk and the resulting cheese (MDWB0 and CDWB0). They were not found in cheeses obtained from the milk of cows fed with wheat bran probably for their inability to grow in the presence of polyphenols. This results confirmed the ability of food polyphenols to selectively modify the growth of LAB [75]. However, the great majority of LAB isolates (almost 98% of total isolates) showed the ability to dominate the microbiota of milk and cheese samples and drive the fermentation process, indicating that DWB diet only slightly influenced the LAB community of cheese.

### 3.6. Economic Costs of Diets and Human-Edible Feed Conversion Efficiency

Table 9 shows the costs of the TMRs consumed by the cows, calculated considering the market prices of each feed recorded during the time of the trial. The cost of the concentrate, hay and total ration decreased significantly with DWB level of the diet, especially due to the lower cost of wheat bran, compared to the other ingredients replaced (barley, faba bean, and feed fat). It is worth noting that the inclusion of more inexpensive feed sources, in this case DWB, in the diet did not cause negative effects on milk production.

The dietary treatment also influenced the heFCE and NFP indexes (Table 9). DWB20 diet reached the best indices, in terms of both crude protein and gross energy, whereas DWB10 diet showed intermediate values. The improvement of the mentioned indexes are in agreement with other studies focused on the effects of different levels of by-products in dairy cows’ feeds [35,51,76,77]. In the present study, the values of NFPcp and NFPge were always negative, due to the considerable presence of corn grains in the rations. With this regard, the higher quality of milk proteins, compared to that of vegetables, should be considered in these calculations, as underlined by Ertl et al. [78].

Therefore, exploiting the ability of cows to convert wheat bran, a by-product used only partially and with low efficiency by humans, into products of high nutritional value, resulted in the improvement of heFCE and NFP indexes. Thus, the use of wheat bran could opportunely contribute to the improvement of the ethical sustainability of animal production which is being increasingly censured when performed in intensive livestock. In this context, wheat bran, as well as other by-products, could be strategically useful to reduce the dependence of intensive farming from cereals and oilseeds used as humans food.

## 4. Conclusions

In this study, feeding the dairy cows with isofibrous diets including wheat bran allowed to achieve the following advantages:

unchanged milk yield,
improved milk quality in terms of protein, casein and curd firmness,
production of cheeses characterized by microbiological characteristics similar to the control cheeses, indicating no effect of wheat bran on the fermentation process,
improvement of the oxidative status and efficiency of immune system of cows,
improvement of the oxidative stability and antioxidant properties of cheese, due to the transfer of phenolic compounds, mainly ferulic acid,
decrease in feeding costs,
improvement of feed conversion efficiency indexes for human food production.

For these reasons, the use of by-products in animal feed formulations is strongly recommended. In particular, due to its adequate composition and polyphenol content, wheat bran was proven to be suitable to counteract the oxidative stress of animals and to increase the content of antioxidants in dairy products. These properties, together with an improved ethical reputation of dairy products on the market, could help to satisfy the increasing demand of modern consumers for food with healthier characteristics and greater sustainability.

## Figures and Tables

**Table 1:** Composition of concentrates (%) and total mixed rations (kg/day per head, TMR) offered to the experimental groups.

	Item	DWB0	DWB10	DWB20
Concentrate	Durum Wheat Bran	0.0	10.0	20.0
	Corn grain	49.5	49.0	50.0
	Barley grain	15.5	13.0	8.0
	Soybean meal	14.5	14.5	14.0
	Faba beans	13.60	7.16	2.36
	Feed fat	2.5	2.3	1.9
	Mineral supplement ^1^	3.90	3.54	3.24
	Vitaminic supplement	0.50	0.50	0.50
Total Mixed Ration	Water	12	12	12
	Hay	11	10.5	10
	Concentrate	14.4	15.00	15.40
	Total	37.4	37.5	37.4

^1^ Calcium carbonate, sodium bicarbonate, sodium chloride, dicalcium phosphate, magnesium oxide, sulfur oxide.

**Table 2:** Chemical composition (% DM), net energy value (Mcal/kg DM) and phenolyc acids content (µg/g DM) of feeds used during the experiment.

Item	Concentrates			Hay	Durum Wheat Bran
	DWB0	DWB10	DWB20		
DM, %	88.75	88.51	88.51	81.99	86.99
Ash	6.87	7.04	6.67	10.46	4.91
Ether extract	2.84	3.14	3.48	1.58	5.14
Crude protein	18.63	18.31	18.07	8.68	16.78
aNDFom ^1^	11.48	13.55	15.72	64.02	36.88
ADFom ^2^	6.26	6.74	6.93	44.74	12.71
ADL ^3^	0.69	1.01	1.27	7.19	3.47
NSC ^4^	59.52	58.17	55.3	15.26	36.28
NE_L_^5^	2.082	2.061	2.059	0.844	1.951
Ferulic acid	1176	1491	1607		1927
Sinapic acid	101	187	219		341
*p*-Coumaric acid	207	190	157		91
Syringic acid	79	120	79		71
Vanillic acid	28	43	38		36
4-Hydroxybenzoic acid	14	16	14		17
Total phenolic acids	1605	2046	2114		2484

^1^ aNDFom = neutral detergent fiber using heat-stable amylase and exclusive of residual ash. ^2^ ADFom = ash-free acid detergent fiber. ^3^ ADL = acid detergent lignin. ^4^ NSC = non-structural carbohydrate = (100 − [CP + Ether extract + Ash + aNDFom]. ^5^ NE_L_ = net energy for lactation.

**Table 3:** Cows’ intake of feeds and nutrients (kg DM/day per head), net energy (MJ/day per head), and phenolic acids (g/day per head) during the trial.

Item	DWB0	DWB10	DWB20	SEM ^1^	*p* Value
Hay	8.80 ^A^	8.40 ^B^	7.85 ^C^	0.026	≤0.001
Concentrate	12.46 ^B^	12.97 ^A^	13.05 ^A^	0.041	≤0.001
Total	21.26 ^A^	21.37 ^A^	20.90 ^B^	0.066	≤0.001
Crude protein	3.08 ^A^	3.10 ^A^	3.04 ^B^	0.010	≤0.001
^2^ aNDFom	7.06 ^B^	7.13 ^A^	7.08 ^B^	0.023	0.066
^3^ ADFom	4.72 ^A^	4.63 ^B^	4.42 ^C^	0.014	≤0.001
ADL	0.71 ^B^	0.70 ^A^	0.73 ^A^	0.002	≤0.001
^4^ NSC	8.76 ^A^	8.82 ^A^	8.41 ^B^	0.027	≤0.001
^5^ NE_L_	139.7 ^B,c^	141.6 ^A,a^	140.3 ^A,B,b^	0.445	≤0.179
Phenolic acids	20.0 ^C^	26.5 ^B^	27.6 ^A^	0.083	≤0.001

^1^ SEM = standar error of mean. ^2^ aNDFom = NDF assayed with a heat-stable amylase and expressed exclusive of residual ash, ^3^ ADFom = ADF expressed exclusive of residual ash. ^4^ NSC = non-structural carbohydrate = (100 − (CP + Ether extract + Ash + aNDFom)). ^5^ NEL = net energy lactation. ^A,B,C^: *p* ≤ 0.01, ^a,b,c^: *p* ≤ 0.05.

**Table 4:** Effect of diet (D) and sampling day (SD) on plasma polyphenols, oxidative status and immune parameter of animals.

Item	Measure Unit	Sampling Day 14			Sampling Day 100			SEM ^1^	*p* Value		
		DWB0	DWB10	DWB20	DWB0	DWB10	DWB20		D	SD	D × SD
Plasma total polyphenols	µg/mL GAE ^2^	120.7 ^b^	155.2 ^a^	147.6 ^a^	147.1 ^a^	151.1 ^a^	160.8 ^a^	6.23	0.005	0.023	0.049
Plasma free polyphenols	µg/mL GAE	30.8 ^b,c^	33.6 ^a,b,c^	27.6 ^d^	23.2 ^e^	29.9 ^c,d^	35.6 ^a^	2.29	0.007	0.452	≤0.0001
Plasma conjugated polyphenols	µg/mL GAE	89.0 ^b^	121.1 ^a^	120.6 ^a^	123.5 ^a^	120.2 ^a^	126.0 ^a^	3.93	0.034	0.015	0.016
ROMs ^3^	U. Carr	108.4 ^c^	93.6 ^f^	102.2 ^c,d,f^	122.2 ^a^	119.0 ^b^	105.4 ^c,d^	5.24	0.028	≤0.0001	0.0159
BAP ^4^	µEq/L	2456	2657	2595	3030	3078	3067	119.2	0.861	0.006	0.928
OSI ^5^	log BAP/ROMs × 100	3.18 ^b^	3.65 ^a^	3.39 ^a,b^	2.88 ^d^	2.95 ^c^	3.31 ^a,b^	0.19	0.041	0.009	0.044
IgG ^6^	g/L	20.03 ^d^	20.03 ^d^	20.02 ^d^	21.34 ^c^	21.41 ^b^	21.63 ^a^	0.01	≤0.0001	≤0.0001	≤0.0001

^1^ SEM = standard error of mean. ^2^ GAE = gallic acid equivalent. ^3^ ROMs = reactive oxygen metabolites. ^4^ BAP = biological antioxidant potential. ^5^ OSI = Oxidative stress index. ^6^ IgG = immunoglobulins. ^a,b,c,d,e,f^: *p* ≤ 0.05.

**Table 5:** Effect of feeding regime on milk composition traits and coagulation properties.

Parameter	Measure Unit	DWB0	DWB10	DWB20	SEM ^1^	*p* Value
Milk	kg/day	25.6	25.7	25.5	1.96	0.981
Lactose	%	4.90	4.98	4.92	0.08	0.578
Fat	%	4.10	4.05	4.01	0.21	0.868
Protein	%	3.51	3.50	3.60	0.10	0.083
Casein	%	2.72	2.71	2.77	0.07	0.084
Whey protein	%	0.72	0.70	0.72	0.006	0.565
NPN ^2^	%	0.032	0.032	0.033	0.0002	0.461
Urea	mg/dL	22.9	21.5	22.3	1.29	0.130
Somatic cells	log10 n/mL	5.22	5.22	5.44	0.40	0.378
pH		6.67	6.69	6.72	0.03	0.198
Titratable acidity	°SH/50	4.00	3.93	3.82	0.09	0.461
^3^ r	min	17.8	18.7	19.6	1.21	0.124
^4^ k_20_	min	3.6	3.3	3.4	0.65	0.765
^5^ a_30_	mm	31.9	36.6	35.3	2.11	0.132
^6^ a_2r_	mm	32.3 ^b^	37.0 ^a^	42.9 ^a^	2.59	0.012

^1^ SEM = standard error of mean, ^2^ NPN = non-protein nitrogen, ^3^ r = Coagulation time, ^4^ k_20_ = Curd firming time, ^5^ a_30_ = Curd firmness, ^6^ a_2r_ = Curd firming after twice r. ^a,b^: *p* ≤ 0.05.

**Table 6:** Effect of feeding regime on cheese composition traits, oxidative products and antioxidant activity.

Parameter	Measure Unit	DWB0	DWB10	DWB20	SEM ^1^	*p* Value
DM	%	68.00	68.31	68.62	2.48	0.693
Fat	% DM	45.11	44.99	44.84	1.91	0.934
Protein	% DM	45.57	45.89	46.32	1.95	0.522
Ash	% DM	5.55	5.57	5.36	0.29	0.795
Total polyphenols	mg GAE ^2^/g	3.65 ^b^	3.73 ^a,b^	4.21 ^a^	0.68	0.095
Number of peroxides	mEq O_2_/kg	1.30 ^A^	1.13 ^A,B^	1.04 ^B^	0.09	0.022
TBARs	µg MDA/kg	4.09	3.95	3.70	0.19	0.501
TAC ^3^	µmol FeSO_4_/g	1518 ^b^	1742 ^a,b^	1848 ^a^	25.6	0.074

^1^ SEM = standard error of mean. ^2^ GAE = gallic acid equivalent. ^3^ TAC = Total antioxidant capacity. ^A,B^: *p* ≤ 0.01, ^a,b^: *p* ≤ 0.05.

**Table 7:** Effect of feeding regime on microbial loads ^a^ of milk and cheese samples.

Samples	Media ^b^							
	PCA-SkM 7 °C	PCA-SkM 30 °C	MRS 30 °C	MRS 44 °C	M17 30 °C	M17 44 °C	KAA	VRBGA
MDWB0	6.21 ± 0.70	6.21 ± 0.80	6.01 ± 0.89	2.95 ± 0.73	6.15 ± 0.98	4.63 ± 0.91	5.18 ± 0.38	4.62 ± 0.78
MDWB10	6.18 ± 0.30	6.70 ± 0.37	5.34 ± 0.82	3.09 ± 0.64	6.89 ± 0.70	4.84 ± 0.94	4.68 ± 0.44	4.69 ± 0.42
MDWB20	6.12 ± 0.33	6.08 ± 0.88	5.32 ± 0.74	3.02 ± 0.53	6.32 ± 0.73	4.55 ± 0.92	4.98 ± 0.65	4.60 ± 0.94
*P* value	0.973	0.569	0.537	0.965	0.541	0.925	0.512	0.987
CDWB0	8.81 ± 0.63	8.95 ± 0.63	7.11 ± 0.63	7.21 ± 0.94	8.81 ± 0.30	8.35 ± 0.69	7.97 ± 0.69	5.78 ± 0.52
CDWB10	8.79 ± 0.24	9.00 ± 0.57	7.37 ± 0.87	7.01 ± 0.81	8.85 ± 0.48	8.08 ± 0.47	7.33 ± 0.45	5.96 ± 0.54
CDWB20	9.01 ± 0.26	9.09 ± 0.28	7.42 ± 0.71	7.10 ± 0.73	8.84 ± 0.50	8.18 ± 0.35	7.49 ± 0.90	5.59 ± 0.74
*P* value	0.783	0.945	0.864	0.958	0.993	0.820	0.546	0.767

^a^ Units are log CFU/mL for milk samples and log CFU/g for cheese samples. Results indicate mean values ± standard deviation (SD) of four plate counts (carried out in duplicate for two independent productions). Abbreviations: MDWB0, control milk, 0% of durum wheat bran, MDWB10, experimental milk, 10% of durum wheat bran, MDWB20, experimental milk, 20% of durum wheat bran, CDWB0, control cheese, 0% of durum wheat bran, DWB, CDWB10, experimental cheese, 10% of durum wheat bran, CDWB20, experimental cheese, 20% of durum wheat bran. ^b^ PCA-SkM 7 °C, plate count agar added with skimmed milk incubated at 7 °C for total psychrotrophic microorganisms, PCA-SkM 30 °C, plate count agar added with skimmed milk incubated at 30 °C for total mesophilic microorganisms, MRS 30 °C, de Man-Rogosa-Sharpe agar for mesophilic rod LAB, MRS 44 °C, de Man-Rogosa-Sharpe for thermophilic rod LAB, M17 30 °C, medium 17 agar incubated at 30 °C for mesophilic coccus LAB, M17 44 °C, medium 17 agar incubated at 44 °C for thermophilic coccus LAB, KAA, kanamycin aesculin azide agar for enterococci, VRBGA, violet red bile glucose agar for *Enterobacteriaceae*.

**Table 8:** Phenotypic grouping of LAB isolated from milk and cheese samples.

Characters	Clusters														
	1 n = 29	2 n = 22	3 n = 18	4 n = 74	5 n = 12	6 n = 11	7 n = 10	8 n = 15	9 n = 55	10 n = 2	11 n = 12	12 n = 4	13 n = 44	14 n = 10	15 n = 11
Morphology ^a^	R	R	R	R	C	C	C	C	C	C	C	C	C	C	C
Cell disposition ^b^	sc	sc	sc	sc	sc	sc	sc	sc	sc	sc	lc	lc	lc	lc	lc
Growth															
15 °C	-	+	+	+	+	+	+	+	+	+	-	-	-	-	-
45 °C	+	-	-	-	-	-	-	+	+	+	+	+	+	+	+
pH 9.2	n.d.	n.d.	n.d.	n.d.	+	-	-	-	+	+	-	-	-	-	-
6.5% NaCl	n.d.	n.d.	n.d.	n.d.	-	+	+	+	+	+	-	-	-	-	-
Resistance to 60 °C	-	-	-	-	+	+	-	+	+	+	-	-	+	+	+
Hydrolysis of:															
arginine	-	-	-	-	+	+	-	+	+	-	-	-	-	-	-
aesculin	-	-	-	+	+	+	-	+	+	+	-	-	-	+	-
Acid production from															
arabinose	-	-	+	+	-	+	+	+	+	+	-	-	-	-	-
ribose	-	+	+	+	+	+	+	+	+	+	-	-	+	+	+
xylose	-	-	+	+	-	+	+	+	+	+	-	-	-	-	-
fructose	+	+	+	+	+	+	+	+	+	+	+	+	+	+	+
galactose	+	+	+	+	+	+	+	+	+	+	+	+	+	+	+
lactose	+	+	+	+	+	+	+	+	+	+	+	+	+	+	+
sucrose	+	-	+	+	+	+	+	+	+	+	+	+	-	+	+
glycerol	+	+	+	+	+	+	+	+	+	+	-	+	-	+	+
CO_2_ from glucose	-	-	+	+	-	+	+	+	-	-	-	-	-	-	-

^a^ R, rod, C, coccus. ^b^ sc, short chains, lc, long chains. Abbreviation: n.d., not determined.

**Table 9:** Cost of the rations (€/day per head) and feed conversion efficiency indexes for human food production.

Item	DWB0	DWB10	DWB20	SEM ^1^	*p* Value
Concentrate	3.65 ^A^	3.60 ^B^	3.39 ^C^	0.15	≤0.0001
Hay	1.00 ^A^	0.96 ^B^	0.89 ^C^	0.08	≤0.0001
Total	4.65 ^A^	4.56 ^B^	4.28 ^C^	0.17	≤0.0001
heFCEcp ^2^	0.54 ^C^	0.66 ^B^	0.72 ^A^	0.02	≤0.0001
heFCEge ^3^	0.51 ^C^	0.62 ^B^	0.69 ^A^	0.03	≤0.0001
NFPcp (g/day) ^4^	−683 ^A^	−496 ^B^	−381 ^C^	4.96	≤0.0001
NFPge (MJ/day) ^5^	−77.8 ^A^	−52.7 ^B^	−39.5 ^C^	1.39	≤0.0001

^1^ SEM = standard error of mean. ^2^ heFCEcp, human-edible food conversion ratio in terms of CP = kg milk CP/kg CP of human-edible feed, ^3^ heFCEge, human-edible food conversion ratio in terms of GE = MJ milk GE/MJ GE of human-edible feed, ^4^ NFPcp (g/day), net food production in terms of CP = g/day milk CP − g/day CP of human edible food, ^5^ NFPge (MJ(day), net food production in terms of GE = MJ/day milk GE − MJ/day GE of human edible food. ^A,B,C^: *p* ≤ 0.01.
